# ‘Far right just means anyone who wants to support British values’: Mobilizing ‘British values’ talk in discussions of the August 2024 UK race riots

**DOI:** 10.1111/bjso.70093

**Published:** 2026-06-10

**Authors:** Rahul Sambaraju, Steve Kirkwood

**Affiliations:** ^1^ Department of Psychology & Neuroscience City St George's University of London Edinburgh UK; ^2^ Department of Social Work The University of Edinburgh Edinburgh UK

**Keywords:** British values, discursive psychology, far‐right, online hate, race riots, UK riots

## Abstract

Social psychological research has shown how far‐right leaders mobilize people by claiming that majority populations are threatened or silenced. This paper builds on this work to examine a related process in naturalistic interactions: how riotous actions are explained and justified through appeals to ‘British values’ in online forums. Using discursive psychology, we analyse talk surrounding the riots that followed the stabbing of three young girls in Southport, England—the UK race riots of August 2024. We show that invoking British or English values serves two key functions: it renders rioters' actions self‐explanatory and offers a competing account of rioters as the more authentic representation of Britishness and the British people than the government. This reframing of ‘British values’ offers up a challenge to those attributing riots to ‘far right’ motivations and instead portrays the riots as effortful and even ideal expressions of British citizenship. Thus, British values operate not merely as symbols but as rhetorical tools that can sanitize the ‘far‐right’ label linking the riots, immigration, state policy and national identity.

## INTRODUCTION

In an interview for The Guardian about the recent initiative to fly the St George's Cross and the Union Jack on lampposts and trees across the UK (mostly England), a ‘flag‐flyer’ named these actions ‘a protest against Westminster^1^’ for not acting in the ‘interests of the British people’ (Boffey, 2025). This form of accounting for actions like Operation Raise the Colours and the worrying rise of anti‐migrant/refugee marches and protests is increasingly offered in terms of efforts to articulate, voice and represent the concerns of the wider, unspecified ‘British people’. In this paper, we examine how British values are drawn upon to account for violent riots across the UK in August 2024.

### British values and dilemmas of belonging

The references to British values are not merely mundane or non‐serious, as one might speak about what makes a nation and its unique character; instead, they hold a socio‐legal importance. David Cameron, as the British Prime Minister in 2014, had made public the importance of ‘British values’ (Wintour, 2014) to combat extremism. What had previously been a loosely defined, aspirational set of norms and codes of conduct shared across different nations was recast as a matter of state policy, particularly in the sphere of public education, and became deeply politicized (Tomlinson, 2015).

Formulated as ‘Fundamental British Values’, these referred to: ‘democracy, the rule of law, individual liberty, and mutual respect and tolerance of those with different faiths and beliefs.’ (UK House of Commons, 2015). However, rather than being straightforwardly embraced, this framework was contentious. Critics argued that it facilitated the othering of Muslims and, more broadly, signalled the erasure of non‐White communities in the UK. Notably, the first official reference to *British values* appeared in the 2011 *Prevent Strategy*—a counter‐radicalization policy widely perceived as disproportionately targeting Muslim communities (Lander, 2018).

Jarvis et al. (2020) examined the wider uptake of the political interest in ‘British values’ in the UK, especially when they were framed as a response to ‘Muslim extremism’ and critiques of multiculturalism. They showed that ‘values talk’ was highly flexible: while participants invoked ideals such as freedom, democracy, tolerance and the rule of law, these were also described as myths concealing intolerance towards migrants and minority groups. British values were sometimes contrasted with those of other nations, particularly Muslim‐majority countries. A later study (Marsden et al., 2023) developed this further, showing how such discourse could stigmatize Muslims as a social ‘danger’. Echoing Condor (2000) and Condor and Abell (2006), participants often expressed discomfort with British values because of colonial associations and highlighted their potential for misuse by politicians and the media to critique immigration or Islam.

The proposed Education (Values of British Citizenship) Bill (Wheeler, 2024) – to teach what it means to be a British citizen in schools – underscores attempts to explicitly link ‘British values’ to citizenship, regardless of its eventual passage. Instead of bearing upon legal requirements for citizenship, the Bill and the wider discourse on British values treat citizenship as more about negotiated moral and civic practices (also see Condor, 2011).

Two features of promoting British values as constituting citizenship stand out: first, polity membership is formulated in ways that elide the problems with treating race or religion as key arbiters. The latter elements can invite accusations of being racist (Augoustinos & Every, 2007; Lynn & Lea, 2003). Second, citizenship is signalled as an active effortful polity membership through practicing these values. Research on citizenship processes shows that belonging is framed through effortful participation, such as seeking employment (Gibson, 2011), legitimizing citizenship claims (Scully, 2024), or justifying exclusion (Gibson & Hamilton, 2011). Studies on citizenship by naturalization highlight emphasis on socio‐political knowledge and practical integration (Andreouli & Howarth, 2013; Gray & Griffin, 2014).

Together then values' talk in relation to British citizenship can *potentially* allow these outcomes. However, this very feature of talk referencing British values or ways of being properly and fully ‘British’ that attracts extremist political actors. The rhetoric of British values can and has been used by political leaders like Nigel Farage, Suella Braverman and Kami Badenoch, and social media influencers like Daniel Niel, to justify harsher migration policies, without seeming to be racist.

### Mobilization and ‘extremism’

Social psychologists have examined how far‐right leaders advance exclusionary politics by portraying minority cultures and Islam as threats, thereby legitimizing discriminatory policies (De Castella et al., 2009; Wood & Finlay, 2008). Such positions, however, are carefully managed: leaders often present themselves as outsiders to the political establishment, victims of ‘political correctness’ or ‘anti‐White racism’, while simultaneously claiming to speak for the ‘ordinary people’ (Johnson & Goodman, 2013; Rapley, 1998; Sakki & Martikainen, 2021; Verkuyten & Nooitgedagt, 2019). Researchers have then turned to examining how the categories of ‘extremist’, ‘moderate’, or ‘marginal’ offer rhetorical resources in presenting their positions as acceptable to many. Hopkins and Kahani‐Hopkins (2009) showed how Muslim leaders in the UK developed competing versions of ‘moderate’ and ‘extremist’ Islam to counter dominant representations, always with Western perspectives in the background. Notably, studies of ‘far right’ politicians demonstrate how figures such as Geert Wilders frame themselves as in the minority and acting for the ‘people’ to counter claims that legitimate politicians must represent the ‘common good’ (Rooyackers & Verkuyten, 2012; Verkuyten & Nooitgedagt, 2019).

Discursive analyses of the far right in the UK have shown how, despite their extremeness, they too might reject being seen as prejudiced (Billig, 1988). Analyses of leaders widely seen as ‘extremist’ similarly show the management of prejudice. Because ‘far right’ discourse often vilifies minorities (Sakki & Pettersson, 2016), leaders are accused of racism. These accusations are turned into resources for claiming victimhood, enabling them to mobilize supporters against the establishment (Durrheim et al., 2018; Rovamo et al., 2024). Goodman (2021) showed how the label ‘far‐right’ was flexibly invoked in Brexit debates to ultimately render the far‐right as a relevant political position. Online platforms further amplify such dynamics: digital tools enhance the rhetoric of the far or radical right (Sakki & Pettersson, 2016). Studies of online accounts involving far‐right groups like the English Defence League show how they may at times align with minorities (e.g., supporting Jews while denigrating Muslims), which they may marginalize at other times (Burke, 2017; Burke et al., 2020).

Given that far‐right positions risk being dismissed as extreme or marginal, politicians and other actors in support of such parties and positions tend to use discourse in ways that normalize the far‐right. For example, Verkuyten and Nooitgedagt (2019) illustrated how the leader of a far‐right political party in the Netherlands presented himself as representing the ‘ordinary people’, whereas other politicians criticized him by portraying him as not fulfilling his democratic duties as a member of parliament. Similarly, Mols and Jetten (2016, p. 289) showed how far‐right politicians legitimized their positions by presenting themselves as on the side of ‘ordinary hard‐working taxpayers’ who were being victimized by immigrants and elites. Sakki and Martikainen (2021) found similar discourse in online election campaigns, where refugees were constructed as threats (including sexist imagery), embedded in stories of a grand past that was now under threat, where elites were the enemy, and right‐wing parties provided the solution. The above findings then show that far‐right political leaders not only construct specific versions of nationhood and national belonging but also their own identities as marginalized yet representative figures. These strategies enable them to legitimize hostility towards racialized minorities and have been implicated in collective violence, including riots (Bagguley & Hussain, 2008).

### Riots, identities and justifications

In one of the earliest examples of social psychological analyses of the discursive construction of ‘riots’, Potter and Reicher (1987) examined the 1980 St Paul's ‘riot’, in Bristol, England, constructions that emphasized pre‐existing problematic relations between the police and the community, in tandem with accounts that portrayed the police as instigating the incident, justified the actions of community members in terms of ‘rebelling’ or ‘fighting back’.

Howarth (2014) analysed social representations in research interviews with people who witnessed the ‘riots’ in Brixton, London, England, 1995, which began after the death of a Black man, Wayne Douglas, while in police custody. Findings showed that framing police as racist and responsible legitimized rioters' actions, while portraying participants as criminals justified police responses. Stott et al. (2018) drew on a range of data sources to explore how ‘riots’ in 2011 in Tottenham and Hackney, England, unfolded and were accounted for by witnesses and those directly involved. Representations of the police functioned to legitimize the actions of those involved, specifically that the police had shot an innocent, young Black man – Mark Duggan – and were using unjustified force against members of the community (Sambaraju, 2022).

Research on the 2011 riots further illustrates how public accounts deploy competing explanations, such as criminality, marginalization or moral collapse, to perform political work (Goodman et al., 2014). Social identity theory approaches emphasize how grievances, policing and collective mobilization interact to produce riot dynamics (Stott et al., 2018). In line with such an approach, Hoerst and Drury (2023) examined online videos relating to the 2017 ‘Unite the Right’ rally and 2021 Capitol insurrection, both in the USA, illustrating the role of collective grievances, senses of growing strength in these movements and the importance of event dynamics. Drury (2024) further explained how political actors capitalize on current events to develop xenophobic narratives. While references to national values are clearly tied up with more general talk about the nation, culture, and claims about desirable ways of being (e.g., ‘Britishness’; see Reicher & Hopkins, 2000), specific references to ‘values’ deserves greater attention given the recent prominence in policy and political discourse.

It is then likely that riots that target ethnic minorities, racialized others and migrant communities will be explained by reference to extremist or far‐right positions. As shown above, those accused of being far right and advocating for those positions have at their disposal rhetorical tools that present them as acting in line with the nation's interests and values. It is this that motivates our present paper, where we examine how references to British/English values are implicated in talk about anti‐migrant riots in the UK. We then take such an examination through a discursive analysis of naturally occurring online discourse about British values in the context of race riots in August 2024, across various cities in the UK.

### The present study

The race riots of August 2024 were a watershed moment in the recent history of race relations in the UK. Ostensibly, these were sparked by the stabbing of three young girls at a dance rehearsal in Southport, England. The perpetrator, although a Black British Christian, was initially rumoured to be a Muslim migrant on social media and other communicative spaces. This led to immediate violence across towns and cities in England and then to other places in Wales and Northern Ireland, causing much damage to lives and property (Osgood, 2024).

Several reports suggest that ethnic and racial minorities were directly targeted, the majority of these being Muslim and Brown persons (Duncan et al., 2024; Jones, 2024). For older adults, the riots reminded them of racist riots in the 1970s and 80s when, Brown and Black people across the UK were frequent targets of both impromptu and organized racial violence (Safdar, 2024). The race riots of 2024 are attributed to far‐right groups and a mobilization of anti‐migrant and racist sentiments in the news media, by politicians and activists. Similar to accounts of riots in other instances these involve the salience of social categories and identities: who participated in these riots? Who were these riots against? The salience of these is to explain, account for and legitimize (or not) the actions of rioters.^2^ In the present case, aspects of being British and British identification alongside ideological affiliation with far‐right groups and ideologies are salient in explicating the actions of rioters. Our interest is primarily in how British/English values as ways to inform being British are salient and used in explaining, justifying and criticizing the riots.

## METHOD

### Data and participants

The data for this study are comments on YouTube to news coverage of the riots in the UK in August 2024. Using a Firefox browser, YouTube was accessed without creating an account, and all data was collected on a single date (20/5/2025). Videos were searched for ‘UK riots news’, resulting in several hits. The following criteria were used to identify relevant videos: published by reputable and widely known news sources (BBC, Sky, Channel 4, ITV, Independent and Telegraph); about the UK riots in August 2024; news coverage and not debates or discussions; in English and have comments. We selected every 3rd video until we reached 10 videos. For each of these, we selected 10 opening posts and inspected all the comments in the thread for these opening posts (in excess of 300 per thread). Comments to these 100 opening posts did not all have further responses. We only selected those instances where responses to the opening posts themselves had responses (i.e., at least three turns), resulting in 100 threads each with at least 20 comments. This was to ensure that the data were sequences of interaction where there was a relatively meaningful engagement.

These were manually collected from YouTube through copy‐pasting onto a MS Word document, preserving the original formatting. This study was approved by the university ethics committee. To mitigate ready identification, we have used pseudonyms for all users to retain a sense of privacy, although interactions on YouTube are publicly accessible and traceable.

Users brought up several aspects in these comments: migration and asylum‐seeking, misinformation and racial relevance. As responses to news coverage, these were topicalized in explaining violence by ‘rioters’ and police, negotiating the role of wider entities like the media or government, and possibilities for the future. However, none of the videos made British / English values explicit, but there were references to Britishness and cultural differences as possible reasons for rioting. For the present purpose, we solely focused on aspects where national values were explicitly topicalized – we did so through the CTRL+F function of MS Word to search for ‘English values’ and ‘British values’. This resulted in 23 salient interactions, which came from 7 videos (14 threads overall and 67 individual comments).

### Positionality

Framing and analysing interactions like these inevitably raise questions around the position of us as authors in describing various agents and positions, like ‘rioters’, ‘protestors’, ‘far right’, ethnonationalism and so on. This is particularly complex given that these terms are also participants' terms. We then want to make clear that we use the term ‘rioters’ for descriptive purposes, albeit with the full acknowledgement that this will imply that they are taking violent or other actions. The first author is of South Asian origin and has experienced racism since moving to Europe, and is of the express view that these riots were racist for targeting Brown and Black persons and institutions. The second author is a Pākehā New Zealander of Irish descent who has been living in the UK for more than 20 years. He has liberal political views and has not personally experienced racism.

### Analytic procedure

The data are analysed using discursive psychology (Edwards & Potter, 1992; McKinlay & McVittie, 2008), especially that which applies to online interactions (Giles et al., 2015; Goodman & Locke, 2024; Sambaraju & McVittie, 2020). We paid attention to the unique resources and affordances of online communication, such as emojis and links to other sources. We approached topically connected online spaces – like comments to YouTube videos on race riots – as polarized ‘digital spheres’ (Sambaraju, 2026) where terms, categories and phrases are imbued with ideological meaning. These meanings rendered interactions not only mutually intelligible, but intelligible in specific ways: as referring to the role of elites, Muslims/Islam‐as‐threat, or Whites‐as‐targets. We were focused on how users oriented to and negotiated such possible inferences through a close examination of how users constructed certain accounts, used categories and responded to other comments to challenge or undermine each other's positions.

In the analysis, we focused on discursive practices by which the riots and related violence were justified, challenged and negotiated, which specifically involved references to ‘British/English values’. We were less concerned with what exactly these ‘values’ were: ideological, behavioural, or dispositional. Instead, the focus was on how the references to ‘British/English’ values functioned in their interaction for the users in their sequential location in responses and comments to news videos on YouTube (e.g., as rhetorical moves to criticize or defend certain positions). We then focused on how these references explained and justified the actions of rioters, managed their stake in explaining and justifying and constructed a rhetorical space in which the actions of the rioters, migrants and the Government are to be understood (Goodman & Locke, 2024). In the analysis, these references were used to reject the categorization of rioters and their actions as ‘far right’, to treat these actions as legitimate and characterize the labelling as ideologically motivated.

## ANALYSIS

Our focus on the use of ‘British / English values’ in talk of riots showed a very interesting phenomenon: talk of values was intertwined with negotiating attributing riots to the far‐right. Participants were negotiating whether to attribute the riots to far‐right persons, groups or ideologies, and it is in these instances that references to ‘British/English values’ come up.

The results are organized along two thematic sections: the first examines extracts where users reference ‘British/English values’ to explain and justify the actions of the rioters. Notably, these justifications appear in rejecting explanations that attribute the riots to the far‐right as individuals, groups or ideologies. The second shows how the reference to British / English values offers a competing account for the rioters' actions in contrast to problematic inferences about the far right. Instead, their actions are constructed as effortful contributions towards supporting the British way of life and as more appropriate representations of the British/English polity than the actions of the government. Together, these downgrade the ascription of the label ‘far right’ in favour of constructing the rioters' actions as effortful and valuable forms of citizenship.

### British values and the ‘far‐right’

Below is a ‘stem’ interaction (Extract 1) between an OP [Original Poster] ‘S73’ and a responder, which is then responded to by other users. In the subsequent extracts, we examine further responses (Extracts 1a and 1b) to this ‘stem’ interaction. This set of comments comes from a news video published by Channel 4 News on August 5, 2024, titled ‘UK riots: 400 arrested amid far‐right violence and clashes with police’.

#### ‘Far right’ and ‘English values’

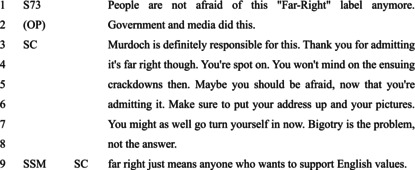

S73 problematizes the use of the ‘far right’ as a category label to describe those rioting, and attempts to sanitize the label. S73, however, does orient to the undesirable aspect of the category ‘far right’. First, S73 treats this merely as a label and puts it in scare quotes to reject its seriousness (Xiong & Robles, 2023). Second, S73 downgrades the severity of social taboos routinely attached to it by indicating a shift in its acceptance: ‘people are not afraid …. anymore’. Third, S73 introduces ‘people’ as stakeholders in the use of this label to reject its problematic status. The category ‘people’ is a broad, apolitical term that functions to present those actively involved in the riot or those who are labelled as ‘far right’ as general members of society, rather than a minority or marginal group (Verkuyten & Nooitgedagt, 2019). Fourth, SC73 introduces ‘Government’ and ‘media’ in contrast to ‘people’ as being responsible for some unspecified outcomes: ‘did this’ (also see Sakki and Pettersson (2016) and Sakki and Martikainen (2021), for analysis on the rhetorical uses of making distinctions between “ordinary ‘good’ people” and “the malicious elite”).

SC in response, however, treats the category ‘far right’ not merely as a label but as having serious real‐world outcomes. These are offered as predicates tied to the category, indicating a normative understanding of ‘far right’, precisely the type of inferences that S73 aims to mitigate (Hester & Eglin, 1997). SC's characterization of the violence as ‘far right’ is attributed to S73's admission, mitigating implications that SC, like others, is merely categorizing others as ‘far right’. SC then introduces the possibility that actions routinely associated with ‘far right’ and responses to those who admit to being ‘far right’ apply to the S73. This takes the form of a series of directives addressing the S73 (lines 5–7): ‘you should be afraid’; ‘make sure to put your address up and your pictures', and ‘You might as well go turn yourself in now’. Together, these directives are hearably ironic (Clift, 1999) because, in admitting to a suspect group membership/affiliation, the expectation is to cover up one's tracks instead of laying bare one's identity, as SC suggests. SC is then making fun of S73 for having admitted to their affiliation with ‘far right’ positions. SC has then rejected the use of ‘far right’ as a non‐serious label and developed the potential problems with being affiliated with this category/position, such as its potential criminality.

Thus far, then, the S73 and SC have offered contrasting constructions of ‘far right’ (cf. Goodman, 2021). It is here that SSM offers another formulation of the category ‘far right’, which rejects any readily problematic association. Specifically, SSM challenges the normative and routine understandings of the term ‘far right’ through characterizing its use as frivolous: ‘anyone who wants to support English values’. The extreme‐case formulated (Edwards, 2005; Pomerantz, 1986) categorization indicates an indiscriminate use of the label ‘far right’ where mere support for Englishness is problematized. In doing so, SSM undermines the normative inferences and predicates that might be mobilized in using the category ‘far right’. Instead, SSM offers a minimal and pointedly (‘just’) simplistic view of being ‘far right’.

This also resonates with S73's claims that it is ‘people’ that are being labelled as ‘far right’ in problematic ways. SSM then explains why apolitical and generic ‘people’ are being categorized as ‘far right’: they are in support of English values (cf. Mols & Jetten, 2016; Sakki & Pettersson, 2016). The comment has a self‐sufficient nature (Billig et al., 1996), as acting in line with ‘English values' inherently implies that such an inclination is appropriate in an English context. Equating these actions as ‘far‐right’ then suggests a serious error in the use of this term, and specifically, an anti‐English position. Below, we examine other responses to SC's construction of normative aspects associated with the term ‘far right’ (hence labelled as 1a).

#### Defending ‘English values’

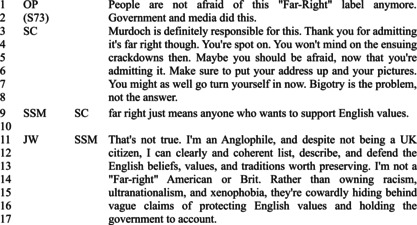

JW rejects SSM's claims that English values can be related to the sorts of violence seen in the race riots. JW, in response, treats SSM as making a truth‐claim (reflexively accounting for JW's engagement) and rejects it. This rejection involves separating an affiliation with England and Englishness from the family of ideological positions similar to far‐right: ‘racism, ultranationalism, and xenophobia’ (lines 14–15). First, JW presents themselves in terms of two sets of possible identities: ‘I'm an Anglophile’ contrasted with ‘I'm not a “Far‐right” American or Brit’. The self‐identification relates to the two categories mentioned earlier by SSM (Widdicombe & Wooffitt, 1995). JW then presents themselves as an example that rejects the category incumbency outlined by SSM. This way of particularizing themselves aligns with a favourable version of being English / Anglophile and subscribing to English values (Billig, 1996).

Second, instead, JW offers another categorization of those who might strategically make references to ‘English values’: ‘cowardly’ (Eglin & Hester, 1999). In contrast to the OP's construction of those involved in the violence as lacking fear, here JW presents the justification of supporting ‘English values’ as both born out of fear and a false pretext for the violence.

Overall, though the implication that English values might be a valid basis for behaviour is maintained, the nature of such values worth defending is distinguished from those supposedly underlying the behaviour of the rioters. This functions to present the rioters as lacking moral standing, dishonest in their justifications and as going against the core characteristics of the nation, which, however, are unspecified (Jarvis et al., 2020). JW then protects Englishness from associations with ‘far right’, without, however, specifying the former.

#### Alternative ‘English values’

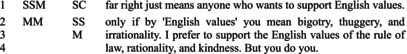

MM's response to SSM bears similarities to the contribution from JW in picking up on the ambiguity of the term ‘English values’, placing it within quotation marks to indicate the potential multiple meanings at play or the way it might be used to indicate something other than the most self‐evident referents. While it takes the form of a concession – ‘only if’ – MM does not offer any agreement (cf. Antaki & Wetherell, 1999). Instead, MM undermines SSM's account by suggesting their argument is ‘only’ valid if the English values referred to are ‘bigotry, thuggery, and irrationality’ (lines 2–3). The three‐part listing (Jefferson, 1990) together indicates a propensity for violence based on prejudice, and so explains the actions of the rioters and presents them as reprehensible.

In contrast, MM offers a contrasting three‐part set of values that are not associated with violence: ‘the rule of law, rationality, and kindness’ (line 4), which overlaps with the Fundamental British Values from the UK Government (UK Gov, 2015). JW and MM then reject the co‐opting of Englishness to justify the actions of rioters and reject their characterization as ‘far right’ or ‘racist’.

#### Using contrasts to defend English values

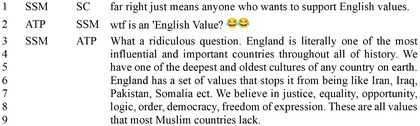

ATP responds to SSM with a question, mocking in tone, that queries the nature of ‘English values’. The use of the initialism ‘wtf’ (What the Fuck) and laughing emojis mocks the reference to English values. The interaction shows similar dynamics to those highlighted by Goodman and Locke (2024), in terms of the use of ridicule, rejection and counterpoints. Here the response from SSM, starting with ‘What a ridiculous question’, functions as an opposing pole to the questioning of English values. This reversal of ridicule indicates that the content of ‘English values’ is rather clear. If ATP portrays English values as something that lacks an obvious definition, SSM treats such values as being self‐evident. SSM's response details the nature of such values, related to rationality and fairness. When SSM presents these as a contrast to the alleged values of several majority‐Muslim countries – ‘Iran, Iraq, Pakistan, Somalia’ – this is made explicit as a shortcoming: ‘most Muslim countries lack’ (Tomlinson, 2015). SSM then develops a ready superiority of being English/British.

While those comparative countries are framed in terms of religion, English values are presented in a secular way, a form of discursive deracialization (Goodman & Burke, 2011) This way of pointing to the value differences between England and other Muslim‐majority countries protects SSM.

### Values, effort and the British exemplar

In this section, we examine instances where users treat the actions of those rioting and the actions of the far‐right as effortful actions taken up in support of British values. This is done to develop inferences that the actions of the far‐right, like protesting/rioting, are better representations of British polity than those of other agents, including the Government. This set of comments in Extract 2a comes from comments to the same video as above.

#### The ‘far‐right’ as a ‘voice of the people’

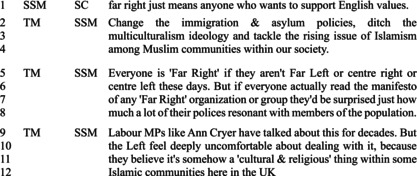

Rather than criticizing SSM's reference to ‘English values’, TM comes in with three contributions that build the case that the ‘far right’ label is facetious and that those who are labelled such are more of a majority than a minority (Verkuyten & Nooitgedagt, 2019). The first contribution identifies immigration, multicultural policies and increasing politicized adherence to Islam in the UK as a source of the problem.

The second contribution, as with other examples, puts the term ‘far right’ in scare quotes to indicate its suspect nature as a contentious category (Eglin & Hester, 1999). This is developed through some boundary work where individuals who are not members in other political groups are ascribed the category “’Far Right’”: “Everyone is ‘Far Right’ if they aren't Far left or centre right or centre left these days”. The use of the extreme‐case formulation (Pomerantz, 1986) (‘Everyone’) along with the temporal marker (‘these days') treats the ascription of the category label ‘Far Right’ as indiscriminate and contextualizes it to the current times.

In their next comment, TM develops the far‐right position as more aligned with the British polity (cf. Sakki & Pettersson, 2016). The next statement, starting ‘If anyone actually read the manifesto...’, is a sweeping statement that portrays many others as failing to deal with the facts of the case. The claim specifically is that the actual policies of those parties that are referred to as ‘right wing’ would resonate with ‘members of the population’ (Mols & Jetten, 2016), a broad term similar to ‘people’ featured in other contributions (Extract 1). This works to legitimize ‘right‐wing’ views as relatively mainstream or acceptable to the British polity. The juxtaposition of legitimating the far‐right as a political position alongside characterizing this position as in line with the British polity treats the actions of the rioters not as an aberration but as an exemplar (cf. Hopkins & Kahani‐Hopkins, 2004).

The final contribution then supplies a reason why this legitimate position is not platformed enough or is politically taboo. TM attributes this to sensitivity to offending Muslims in the UK (Lander, 2018). Notably, the supposed appeasement itself is treated as the problem for suppressing pride in English values (Tomlinson, 2015).

Below, users are negotiating whether the actions of the rioters represent British values. This interaction was taken from comments to a video published by Sky News on August 7, 2024, titled: ‘UK riots: Britain braced for more than 30 protests across towns and cities’.

#### Far‐right and care for the country

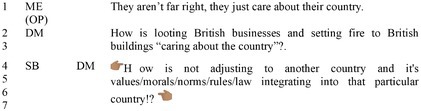

DM challenges claims that the actions of rioters were in the interests of the UK, as claimed by the OP (ME). ME's post itself is presented as a challenge to broader claims that the rioters are ‘far right’ and instead attributes their actions to ‘care for their country’. This disposition is hearably normative and expected – a ‘citizen’ can be legitimately expected to care for their ‘country’ (Hester & Eglin, 1997). In dealing with the possible equivalence between a normatively expected ‘good’ action towards one's nation with the problematic label ‘far right’, ME offers it up as a puzzle to be solved.

DM's challenge is then based on characterizing the actions of the rioters, as ‘looting’ and ‘setting fire’ to British entities – ‘buildings’. These activities are normatively tied to categories like ‘looters’ or ‘vandal’ (Eglin & Hester, 1999). However, compounding these actions with the national category ‘British’ treats the members in these categories and these actions as anti‐British, instead of what ME had suggested, which is to act in British interests.

To this, SC offers a ‘what‐aboutery’ (Finlay et al., 2024) form of response, insofar as it offers another set of questions that echo a similar paradox. The paradox is a contrast between migrants' claims of ‘integration’ while apparently not ‘adjusting’ to the ‘values/morals/rules/law’. Notably, SC does not make explicit the UK and instead offers this in generic terms – ‘another country’ and ‘particular country’ – to treat the expectation that migrants need to adjust as a *norm* instead of a uniquely British expectation. SC then indicates that expecting migrants to adopt British values is unproblematic, and so it is migrants' fault for not putting in the effort in following or subscribing to a set of interchangeable value‐extensions: ‘morals/rules/law’ (see Scully (2024) for a discussion on how effort is used to negotiate social citizenship in Ireland). SC then heads off complaints about rioters with complaints about migrants, suggesting that perhaps the actions of rioters are not in doubt vis‐à‐vis allegiance to the nation, and instead it is migrants who are suspect for not showing normatively expected effort. Below, the element of effort takes a different form, contrasting British persons' effort with that of asylum‐seekers.

#### Regular’ British person versus ‘threating’ migrant

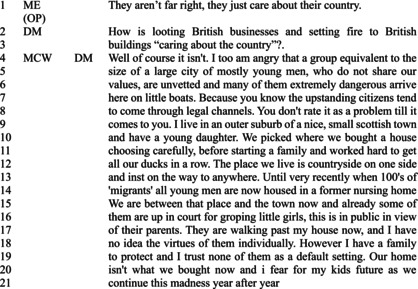

Above, MCW offers another challenge to DM. This, however, is couched in treating DM's post as asking pointed questions. MCW's opening response ‘it isn't’ treats DM as having offered an interrogative (Raymond, 2003). The relevance of this is that it treats DM as proceeding from the premise that the rioters' actions are aimed against British establishments, and so downgrades their citizenship or allegiance to the UK. MCW describes migrants in categorial and dispositional terms: ‘young men’, who ‘do not share our values’, ‘unvetted’, ‘extremely dangerous’ and ‘arrive here on little boats’ (lines 5–7). These constructions draw upon common problematic tropes of asylum‐seekers and refugees to collectively suggest illegitimacy (Goodman & Locke, 2024; Leudar et al., 2008; Lynn & Lea, 2003).

MCW explicitly indicates that their ‘anger’ is not merely about migration or migrants but is grounded in the personal relevance of the problem with migration: ‘till it comes to you’. This is done through describing the effects of this migration on their locality – ‘outer suburb of a nice, small Scottish town’ (line 9) – and the composition of their ‘family’: ‘have a young daughter’. MCW attributes their position to effortfulness – ‘worked hard to get all our ducks in a row’ – indicating favourable features. The juxtaposition of the description of migration and MCW's idyllic life suggests the potential for disturbance through elements of gender, illegality/criminality, numbers and propensity for danger.

MCW's subsequent descriptions suggest the realization of these fears: ‘already some of them are up in court for groping little girls’ (lines 15–16). Further, their descriptions of proximity to MCW's home, and by extension to their ‘young girl’, construct the presence of migrants in their area as giving rise to fear for those such as MCW and their family. The use of gendered categories matched with descriptions of danger and inappropriate sexual behaviour, with an implicit racial dimension, does significant category work (Stokoe, 2012), whereby young migrant men are characterized as violent sexual predators who pose threats to women and children (e.g., Sakki & Martikainen, 2021). While this is hearable as stereotyping (Whitehead, 2011), MCW is careful to account for characterizing migrants as possibly deviant and criminal: ‘I have no idea the virtues of them individually’ and ‘I trust none of them as a default setting’ (lines 18–19). In doing so, MCW manages their stake (Potter, 2010) by conceding that they are generalizing and that this is problematic (Antaki & Wetherell, 1999), but defends this perspective on migrants through foregrounding concern and care for their family: ‘I have a family to protect’ and ‘I fear for my kids’ future’. Alongside claims about how migrants disturb their regular life, the implicit suggestions about their hard work in contrast to migrants' possibly perverse or not ‘upstanding’ aspects, downgrades migrants' status as regular and effortful potential citizens (Gibson & Hamilton, 2011; Scully, 2024). These constructions are ascribed more laudable values to MCW in contrast to problematic values and behaviours to migrants.

Below, similar concerns with the potential criminality of migrants are used to make the claim that rioters were acting in favour of British values, but are persecuted by the State. The extract below includes comments taken from a video published by Sky News on August 16, 2024, titled ‘UK riots: Do prisons have the space to jail rioters?’

#### The far‐right as Bona fide British citizens

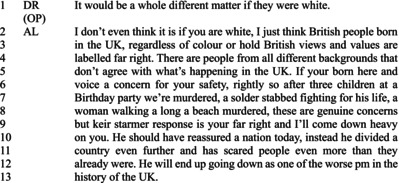

The OP has made race salient in pointing to different outcomes based on race. While it is not explicit, in the context of the video and other comments, we assert that the OP's claim is about how policing of non‐White communities is softer. This claim, often referred to as ‘two‐tier policing’ (Brown, 2024), is a routine complaint among far‐right activists (Chouliaraki & Higgings, 2025).

AL's response, while seemingly disagreeing, furthers OP's claims about how the police or the Government respond to those protesting. Instead of attributing ‘two‐tier’ policing to racial preference, AL develops the claim that the Government and police target those who are quintessential British persons: ‘hold British views and values’ (Chouliaraki & Higgings, 2025). Notably, AL rejects race as a factor in how the Government might respond and offers a formulation of those targeted based on nationality: ‘British people born in the UK, regardless of colour’ (lines 2–3) (Goodman & Burke, 2011). This is a much more serious concern, since it is normative that the Government is aligned with the concerns of its citizens, in this case, the British people upgraded as quintessentially British (for upholding British values). This claim is bolstered in the following ways:

First, AL develops the bona fide status of those being targeted while rejecting categorizing them as ‘far‐right’. This is done through ascribing to them actions in support of the wider British polity (Reicher & Hopkins, 2000). AL eschews possible implications that these are White persons, strengthening their claim that protests are less about racial grievance and more about national interest: ‘There are people from all different backgrounds that don't agree with what's happening in the UK.’ (lines 4–5).

Second, AL constructs these acts or protests as legitimate by presenting them as a response to other problematic events. The three‐part listing (Jefferson, 1990) of these events, and the variety of targets and everyday activities in which they were involved, function to present the risks as applying to the wider British polity. AL explicitly marks responses to these acts as legitimate: ‘rightly so’ and ‘genuine concerns’ (Lynn & Lea, 2003). Here, AL does not offer any indication about the perpetrators, but it is widely known that these were perpetrated by persons with a migrant background. In not making this explicit, AL has protected themselves from being seen as racist or xenophobic (Goodman & Burke, 2011).

Third, then, AL reports British Prime Minister (PM) Keir Starmer's response to voicing what are presented as genuine concerns, as ‘far right’, which is hearable as contrasting with the genuine concerns of the British people. AL makes this explicit in drawing a contrast between what a PM ought to have done – ‘reassure a nation’ – and the outcomes of labelling those protesting as ‘far right’: ‘divided a country even further and has scared people even more than they already were’. The undermining of Starmer's actions is not merely a complaint directed at the personal failing of the PM, but is developed in terms of failing to act as an elected representative of the British people (cf. Verkuyten & Nooitgedagt, 2019). Notably, it is not merely that Starmer is ‘elite’, but that there is a failure of duty towards the British people (Sakki & Martikainen, 2021). The implicit contrast is with the upstanding British citizens who are acting in the interests of the British polity – ‘far right’.

In the last of the extracts examined here, users take a jocular stance towards these ‘effortful’ actions by those rioting. Despite the jocularity, the actions of the rioters as effortful or not are considered for informing rioters' relationship to British values. The extract below includes comments taken from a video published by The Telegraph on August 6, 2024, titled: ‘The aftermath of riots in Hull’.

#### Irony, humour and values in downgrading ‘riots’

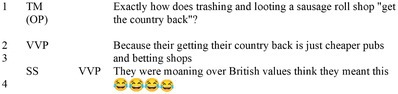

Above, the OP points to the strangeness in damaging a fast‐food shop (‘sausage roll’) to achieve nation‐relation objectives: “get the country back”. The use of scare quotes points to the odd or ironic nature of such objectives (Billig, 2001). Notably, the action description of protests and riots is downgraded, say from protesting for the rights of British persons to that of ‘trashing’ and ‘looting’.

VVP orients to this as ironic and offers two trivial items as informing ‘getting their country back’: ‘cheaper pubs’ and ‘betting shops’. These items relate to the item included in the OP's post – ‘sausage roll shop’. Of note is that these spaces are associated with leisure and not necessarily with what might be routinely understood as productive. This transforms the actions of the rioters from claims about being in the service of the country to those of frivolity. Further, there is a possible pun here insofar as ‘value’ is taken to mean in monetary terms, and so rioters' actions are in support of a better value for their money. Nevertheless, VVP and TM both make salient elements of effortfulness in denying the legitimacy of rioters as quintessential British persons. SS takes up this inference and offers a jocular response that downgrades those rioting (Goodman & Locke, 2024). Specifically, the target of the joke is the avowed attribution of these actions to ‘British values’, when these point to trivial matters.

## DISCUSSION

Reicher (2001) has argued that there is no single collective identity that can be unproblematically applied to those rioting (also see Bagguley & Hussain, 2016). In this paper, we have not attempted to do so. Instead, we have examined how riots, riotous persons and the targets are categorized, and riots explained, in a specific discursive setting: comments to the news coverage of race riots in August 2024 in the UK on YouTube. Justifying race riots faced a ready problem: these were seen as motivated by far‐right or similarly extreme persons, groups and ideologies. Explaining riots as arising from far‐right persons, groups, or ideologies would mean that these actions were racist and damaging to societal well‐being. It is to counter these that references to British / English values were mobilized. Certain constructions of British / English values allowed users to sanitize the label ‘far right’, and upgrade those rioting (even if called far‐right) as better representativeness of Britishness than the majority of the political actors and the Government. Users offered constructions of British values to denigrate the nature or culture of racially minoritized ‘others’ to counter which other actors put forth alternative versions of British / English values that emphasized respect towards people of diverse backgrounds, which functioned to discredit those involved in the riots.

### Managing the ‘far‐right’ label

Much of previous research has shown that speakers would attempt to manage and reject being seen or categorized as prejudiced, anti‐migrant, or far‐right (Billig, 1988; Burke et al., 2020; Goodman, 2021). In the present case, users faced this issue because a ready explanation for race riots is prejudice or far‐right ideology. This was managed not just by rejecting the ascription of ‘far right’ as a label but through whitewashing the category far‐right: this was equated to acting in support of British/English values (Extracts 1–3). Doing so accrued several rhetorical gains for users.

First, this inverted the moral evaluation of the far‐right: instead of being seen as hateful for targeting minorities, the far‐right was cast as standing up for the nation's values. Second, users could offer alternative psychological motivations and reasons for rioting (Edwards, 2003): instead of bigotry, users offered support for and ‘wanting’ to support British/English values (Extracts 1, 1a, 1b and 2). Third, it posed a challenge for other users who might ascribe the race riots to far‐right ideologies. Other users then offered alternative ways to disassociate British / English values from those of far‐right positions. For instance, in Extract 1a, JW rejects SSM's conflation of English values with far‐right positions, while in Extract 2, SSM presents English values positively by contrasting them with those of Muslim‐majority countries. Such framings position Britishness as inherently moral and righteous.

While prior research highlights the far‐right's struggle for legitimacy (Durrheim et al., 2018; Rooyackers & Verkuyten, 2012), here ordinary users—not political actors—worked to normalize the far‐right and anti‐migrant sentiment by aligning it with mainstream British polity (Extracts 2a, 4). As justifications for riots and rioters' actions, this alignment normalizes complaints about and a rejection of migrants and migration. Migrants, particularly from Muslim‐majority countries, were often portrayed as culturally incompatible with Britishness (Marsden et al., 2023). TM (Extract 2a) depicts rioters as reflecting majority sentiment, with politicians positioned as ‘out of touch’ (cf. Rooyackers & Verkuyten, 2012). Some of this aligns with previous research on far‐right discourse, where a pro‐migrant elite is depicted in opposition to ‘ordinary people’, and as threatening core aspects of society, while managing accusations of racism (Mols & Jetten, 2016; Sakki & Martikainen, 2021; Sakki & Pettersson, 2016). Here, however, the key outcome is to rescue the category ‘far right’ from being seen as malicious and hateful towards migrants and ethnic minorities.

### Rioting and effortful citizenship

The findings here also point to the connections between collective action in the form of protests and riots and their justifications by reference to British values and British interests. Rioters' actions were constructed as active efforts in the service of promoting British values, protecting Britishness and representing the British polity. Although equating the far‐right with national identity might seem paradoxical, users achieved this by (1) upgrading the far‐right over other representations of British polity, such as the majority of political parties and the Government (Extracts 2a and 3) and (2) ascribing to the rioters a sense of active effort in preserving British values (Extract 3a, 4 and 5).

Recent scholarship on citizenship has pointed to the relevance of effort in negotiating social and, at times, the legal aspects of citizenship (Gibson, 2011; Gibson et al., 2018; Scully, 2024). This scholarship has focused on those from whom social or legal citizenship is in doubt. Here, however, rioters were constructed as those who were taking up actions because relevant others, like mainstream politicians, political leaders, or the State, failed to do so. It is in this light that rioters come to be cast as exemplars of British person. Users here treated rioters' actions as representing the wider British polity. Instead of being motivated by personal views, extreme ideologies, or dislike of the migrant other, those rioting were constructed as voicing the wider concern of the British polity. It is such actions that were defended and justified, and we argue are indicative of effortful citizenship.

The analysis shows that framing far‐right as merely supporting British values afforded unique rhetorical advantages: one was to recast rioters' actions as in the interest of the British polity and so that categorizing them as ‘far right’ was facetious and two, that their actions were purposeful in protecting Britishness from the migrant threat. Not only are migrants treated as not‐so‐effortful (Extracts 3a, 4) (Scully, 2024), but riots and protests are constructed as effortful actions in the interests of British values: the rioters are closer representations of the interests of the British polity than other political actors (Extract 2a). This is not to suggest that British values are inherently anti‐migrant. Indeed, as the data show, several users offer inclusive and otherwise favourable versions of British values (Extract 1a, 1b, 2). However, when the far‐right is framed as representing the national majority and acting in support of and for the promotion of British values, then their actions cannot easily be marked as problematic.

What is achieved is the rejection and sanitization of the ‘far right’ label: users offered a combined formulation that at once pointed to the inappropriateness of the label and offered an alternative motive for rioting, namely care towards and support for Britishness, formulated as ‘British values’. This form of soft nationalism (Billig, 2005) implied an innocence and righteousness for acting in defence of one's nation instead of outright superiority or pride (however, see Tomlinson, 2015).

What is then novel here is that these attempts to construct the far‐right as exemplars of being British allowed for an indifference towards being categorized as the far‐right. Transgressing this social taboo of not wanting to be seen as racist, extremist, or far‐right problematically translates to the loss of a significant means of stemming anti‐migrant hate: calling out actions, positions and individuals as far‐right.

### Limitations and future research

While the dataset was extensive, references to British or English values were concentrated around a few videos and interactions (14 threads overall and 67 individual comments). None of the videos made British / English values explicit; however, there were references to what it means to be British and cultural differences as possible reasons for rioting. Nevertheless, such references are increasingly visible in political discourse, media commentary and everyday talk. Political figures such as Nigel Farage, Suella Braverman and Kemi Badenoch, as well as commentators like Dean Neil, routinely frame migrants' supposed non‐adherence to British values as grounds for exclusionary policies. Future research should examine these elite discourses and consider whether alternative invocations of British values might counter anti‐migrant sentiment and promote social cohesion.

## AUTHOR CONTRIBUTIONS

**Rahul Sambaraju:** Conceptualization; methodology; investigation; data curation; formal analysis; visualization; writing – original draft; writing – review and editing. **Steve Kirkwood:** Conceptualization; data curation; formal analysis; visualization; writing – original draft; methodology; investigation; writing – review and editing.

## FUNDING INFORMATION

No funding was involved in the preparation of this manuscript.

## Data Availability

The data that support the findings of this study are available from YouTube. These data were derived from the following resources available in the public domain: https://authorservices.wiley.com/author‐resources/Journal‐Authors/open‐access/data‐sharing‐citation/data‐sharing‐policy.html.

## Footnotes

^1^
A common way to refer to the UK Parliament 2024.

^2^
We are fully aware that the categories that we as researchers use are not ‘neutral’ but carry with them moral inferences. Here the category ‘rioters’ for instance.
